# Use of outpatient and inpatient health care services by occupation—a register study of employees in Oulu, Finland

**DOI:** 10.1186/s12913-022-07970-y

**Published:** 2022-05-03

**Authors:** Hanna Rinne, Mikko Laaksonen, Jenni Blomgren

**Affiliations:** 1 The Social Insurance Institution of Finland, P.O. Box 450, 00056 KELA, Helsinki, Finland; 2 The Finnish Centre for Pensions, 00065 ELÄKETURVAKESKUS, Helsinki, Finland

**Keywords:** Outpatient care, Inpatient care, Use of health care services, Occupation, Socio-economic status

## Abstract

**Background:**

The aim of this study was to examine how the use of outpatient and inpatient health services differs by occupational groups, and whether the differences are explained by sociodemographic factors and health status.

**Methods:**

We used register-based data on 25–64-year-old employees living in the city of Oulu, Finland, in 2018 (*N* = 61,848). Use of outpatient health care services (public, private and occupational health care) among men and women was analysed with negative binomial regression models, and use of inpatient health care with logistic regression models, using two occupational classifications: occupational group (1-digit level) and more detailed occupation (2-digit level). Adjusted covariates were age, education, income, marital status, special reimbursement entitlements for medicines, and sickness absence.

**Results:**

Examined at the level of larger occupational groups, the use of outpatient and inpatient health care was less common than average among managers, professionals and skilled agricultural, forestry and fishery workers; in women also among craft and related trades workers. Controlling for covariates explained only part of the differences, more among women than among men. Analysed at the level of more detailed occupations, the adjusted use of outpatient and inpatient care was more common among health associate professionals and stationary plant and machine operators, both among men and women. Furthermore, the use of outpatient care was common among male personal care workers, protective service workers and metal, machinery and related trades workers as well as among labourers in mining, construction, manufacturing and transport, and female customer services clerks and sales workers.

**Conclusion:**

The use of health care services differs by occupation, and the differences are not fully explained by sociodemographic factors and health status. High occupational risks, attitudes and knowledge may explain the more frequent use of health services. Furthermore, explanations may be sought from lack of access to occupational health care or healthier working conditions and behavior.

**Supplementary Information:**

The online version contains supplementary material available at 10.1186/s12913-022-07970-y.

## Background

Socio-economic differences in health have been observed between occupational groups [1] but variation also exists between specific occupations [2–5]. The most important factors behind such differences are material living conditions, social and psychosocial factors, health behaviors and the use of health care [6]. In Finland, health differences between socio-economic groups are comparatively large [2], and part of these differences may be related to unequal use of health care services. Indeed, although the Finnish health care system is based on the principle of horizontal equity [7], Finland ranked poorly in the international comparison of the equal allocation of health services [8].

Several factors may influence the use of health services. On the demand side, individuals seek treatment depending on such factors as their perceived need, earlier experiences of the services, and attitudes towards health care institutions. On the supply side, availability of services, costs and waiting times may affect access to care [9]. Despite the principle of horizontal equity, there may still be differences between occupations in the use of health services. Such differences may relate to, among other things, work-related risk factors [10–12], socio-cultural norms [13] and access to services [14, 15]. Occupational differences in the use of health care services may thus arise from differences in supply and demand factors, which are connected also to socio-demographic factors such as education and income. By controlling for socio-demographic factors and health status, it may thus be possible increase understanding of occupational differences in health care services use.

Health care may be organized as either outpatient or inpatient care. Previous studies from various Western countries have found that the use of outpatient health care services is distributed differently according to occupation-based socio-economic class [16–22]. Due to differences in health care systems, socio-economic classifications, and study designs, previous studies have reached partly contradictory results. Some studies have found that those with a lower position use more outpatient primary health care [17, 21, 22] while others have found no consistent differences between socio-economic groups [18, 19] or no clear pattern [16, 20]. In contrast, specialised outpatient care has been found to be overused in the well-off groups [17, 20–22]. Research on differences in outpatient health care services use by detailed occupation is scarce.

Studies on the use of inpatient care services by occupational class have found increased need-adjusted risk among lower socio-economic classes [19, 21], among upper classes [20] or no difference [17, 21, 23]. At a more detailed occupational level, a Finnish study found that occupations entailing heavy manual work and difficult working conditions had higher risk for inpatient care [24]. Among the six largest occupations, nurses had the highest incidence, while teachers had the lowest [25].

Overall, research on differences in health care services use between occupations, as well as explanations for these differences, is scarce. Most previous studies are surveys with self-reported data and imprecise occupational classifications, and only few studies have used more reliable register-based data. In addition, differences within broad occupational classes, i.e. by detailed occupation, have not been studied. These differences may at least partly arise from socio-demographic factors and health status that vary by occupation. Earlier studies have adjusted for need using self-rated health, chronic diseases or both, whereas only few studies have adjusted also for other covariates such as education [16, 19], income [16, 23] or marital status [18].

We add to the previous literature by studying the use of both outpatient and inpatient health care services by occupation using two levels of occupational classifications. Thus, the aim of this study was to examine how the use of outpatient and inpatient health services differs by broad occupational groups and by detailed occupations among Finnish working-age employees, and whether the differences are explained by socio-demographic factors and health status.

## Methods

We used extensive register-based data on 25–64-year-old persons who lived in the city of Oulu, Finland, in 2018. Oulu is located in the region of North Ostrobothnia and is the fifth most populous city in Finland with 200 000 inhabitants [26]. The data were collected as part of a research project concerning the use of services and social security benefits among the inhabitants of Oulu [27]. The dataset includes individual-level information that is not commonly available in registers, such as comprehensive information on occupational health care services use. We obtained individual-level register data from the City of Oulu, the Social Insurance Institution of Finland, the National Institute for Health and Welfare, the Finnish Center for Pensions, four large occupational health care providers, Statistics Finland, and the Finnish Tax Administration [28]. Socio-demographic data as well as information on the use of social and health services and benefits among Oulu residents were linked from the above-mentioned registers by using personal identity numbers assigned to each Finnish citizen. We restricted the analysis to those who were employees at the end of 2017 and who had worked most of the year, that is more than half of the year 2018 according to the Finnish Center for Pensions’ earnings register (*N* = 61,848).

### Outcomes

We constructed two separate outcome variables, i.e., the use of outpatient and inpatient health care services. The use of outpatient health care was measured by the number of doctor or nurse visits in outpatient primary or specialized health care during year 2018. Finland has a three-sector healthcare system, which includes the public and private sectors as well as occupational health care. Public sector services are available to everyone, but waiting times can be long and there is usually a small customer fee. Occupational health care services are free of charge for their users and access is often fast, but these services are only available to the employed. Private sector services are available with shorter waiting times for those with the ability to pay. Being able to analyse the three sectors together is exceptional, because occupational health care is not covered in registers [8].

Data on public sector outpatient primary health care visits were obtained from the registers of the City of Oulu, while data on outpatient specialised health care were acquired from the Care Registers for Health Care (HILMO). Data on occupational health care visits were derived from the four largest occupational health care providers in Oulu, estimated to cover over 90% of the occupational health care customers [28]. Information on private sector doctor visits was obtained from the registers of Social Insurance Institution of Finland, which reimburses part of the fee charged by a private doctor. We included actual face-to-face visits to a doctor or a nurse; thus, other contact types such as phone calls or emails were not included. Also, visits related to oral health care were not included. As separate visits during the same day were inconsistently recorded in the registers of the different register holders, we approximated the number of visits by contact days with each health care provider. Finally, we calculated the total yearly number of visits (i.e. contact days) in public, occupational and private health care to form the first outcome.

Data on inpatient care were derived from the registers of the City of Oulu and the Care Registers for Health Care (HILMO), including admission dates from both primary and specialized care [29]. We measured inpatient health care as a dichotomous variable, classifying persons to those who were hospitalized at least once and those who were not hospitalized during year 2018, following the procedure used in previous studies [24, 25]. Inpatient care involving rehabilitation, oral health or childbirth was not included. Information on both outpatient and inpatient care could include also visits that took place outside the city of Oulu.

### Occupation

Occupation at the end of year 2017 was obtained from the registers of Statistics Finland. We classified the occupations using the Finnish version of the ISCO-08 classification [30], which consists of five hierarchical levels. We studied the differences between all occupational classes at the 1-digit level. Furthermore, to gain a more profound understanding of the occupational differences, we examined the 20 largest occupations at the 2-digit level. The classification of the 20 largest occupations was made separately for men and women. The 20 largest occupations covered 87% of employed men in the study population and 95% of women, respectively. The rest of the occupations were grouped together (results not shown).

### Covariates

We controlled for sociodemographic factors and two indicators of health status, which, according to earlier studies [31], may influence the use of health services. The sociodemographic factors were age, education, income and marital status. Age at the end of year 2017 was categorized into five-year age groups. Education was based on the highest completed degree or certificate. Those with primary level have completed up to 9 years of education, those with secondary education 11–12 years, and those with tertiary education at least 13 years. Income was measured as personal taxable income during the year 2017 (gross earned income, benefit income and income from capital combined, before taxes). It was classified into quartiles, separately for men and women. Marital status was classified into married, never married and separated/widowed. Income was retrieved from the registers of the Finnish Tax Administration, the other sociodemographic factors from Statistics Finland.

We measured health status through two variables available in the registers: the special reimbursement entitlements for medicine expenses and by sickness absence in 2017. The entitlements to special reimbursement for medicine expenses are available for medicines used in the treatment of severe and long-term diseases, and the information is often used as a proxy of chronic diseases [32]. Persons were classified as having at least one chronic disease if they had any entitlements in 2017. Sickness absence was measured by sickness allowance recipiency. It is available after completing a 10-day waiting period and requires a medical certificate. We added up the sickness allowance days in 2017 and categorized them into 0, 1–60 and 61–365 days. The information was obtained from the register of the Social Insurance Institution of Finland.

The distributions of the sociodemographic factors and measures of health status used in this study by occupational groups (1-digit level) and occupations (2-digit level) are presented in Table 1 for men and in Table 2 for women. Supplementary Table 1 shows the average number of outpatient care visits and the proportion of persons using inpatient care services according to the covariates.

**Table 1:** The distribution of sociodemographic factors and proxies for health status by occupational group and occupation among male employees

	*N*	%	Mean age	Tertiary education %	Highest income quartile %	Married %	Chronic diseases %	Sickness absence* %
**Occupational group (1-digit level)**								
1 Managers	1415	4.6	47.8	84	90	80	19	5
2 Professionals	10,197	32.9	42.7	87	42	60	17	5
3 Technicians and associate professionals	5618	18.1	41.9	66	21	54	18	8
4 Clerical support workers	1067	3.4	41.0	34	6	41	21	10
5 Service and sales workers	3340	10.8	39.8	17	5	42	17	9
6 Skilled agricultural, forestry and fishery workers	96	0.3	43.6	14	4	41	22	14
7 Craft and related trades workers	4670	15.1	40.6	7	6	43	16	10
8 Plant and machine operators, and assemblers	3493	11.3	42.1	10	13	45	19	12
9 Elementary occupations	1079	3.5	39.3	14	2	35	17	11
**Occupation (2-digit-level, 20 most common occupations)**								
13 Production and specialised services managers	721	2.3	47.6	87	90	79	20	5
21 Science and engineering professionals	3995	12.9	42.5	92	46	61	15	4
22 Health professionals	642	2.1	42.1	92	76	64	17	6
23 Teaching professionals	1629	5.3	46.1	85	30	65	21	6
24 Business and administration professionals	1021	3.3	43.4	81	48	63	19	6
25 Information and communications technology professionals	2426	7.8	40.0	82	37	54	15	4
31 Science and engineering associate professionals	2271	7.3	41.5	72	22	53	17	7
32 Health associate professionals	548	1.8	40.3	82	5	54	18	14
33 Business and administration associate professionals	1758	5.7	43.8	63	31	60	19	6
34 Legal, social, cultural and related associate professionals	572	1.8	41.4	42	6	45	20	11
51 Personal service workers	943	3.0	42.0	10	2	42	20	9
52 Sales workers	1414	4.6	38.4	24	9	42	16	7
53 Personal care workers	493	1.6	40.7	12	2	37	21	12
54 Protective services workers	490	1.6	38.9	14	4	49	15	13
71 Building and related trades workers, excluding electricians	2107	6.8	40.0	6	6	46	15	10
72 Metal, machinery and related trades workers	1423	4.6	41.7	6	5	43	17	11
74 Electrical and electronic trades workers	884	2.9	39.8	9	9	40	17	9
81 Stationary plant and machine operators	1103	3.6	42.3	15	27	47	19	14
83 Drivers and mobile plant operators	1968	6.4	41.9	7	6	44	19	11
93 Labourers in mining, construction, manufacturing and transport	630	2.0	39.6	13	3	35	19	13
** All**	30,975	100.0	41.9	50	25	52	18	8

^*^At least one sickness absence day

**Table 2:** The distribution of sociodemographic factors and proxies for health status by occupational group and occupation among female employees

	*N*	%	Mean age	Tertiary education %	Highest income 4th %	Married %	Chronic diseases %	Sickness absence %
**Occupational group (1-digit level)**								
1 Managers	453	1.5	48.7	94	87	69	21	11
2 Professionals	9307	30.1	43.6	95	55	60	20	10
3 Technicians and associate professionals	8469	27.4	43.5	83	18	54	22	16
4 Clerical support workers	2420	7.8	44.7	62	11	50	27	13
5 Service and sales workers	7270	23.5	42.1	18	4	46	22	17
6 Skilled agricultural, forestry and fishery workers	66	0.2	39.2	36	5	39	12	11
7 Craft and related trades workers	352	1.1	41.0	19	7	42	19	10
8 Plant and machine operators, and assemblers	637	2.1	43.4	17	18	43	23	16
9 Elementary occupations	1899	6.2	45.9	10	1	46	25	20
**Occupation (2-digit-level, 20 most common occupations)**								
21 Science and engineering professionals	1013	3.3	41.0	94	55	55	16	8
22 Health professionals	1438	4.7	44.0	97	81	66	21	10
23 Teaching professionals	4073	13.2	44.3	96	47	63	21	11
24 Business and administration professionals	1214	3.9	43.8	92	57	57	22	8
25 Information and communications technology professionals	448	1.5	42.2	91	60	58	19	10
26 Legal, social and cultural professionals	1121	3.6	43.1	93	41	54	20	11
31 Science and engineering associate professionals	595	1.9	41.8	64	28	49	20	9
32 Health associate professionals	3943	12.8	43.2	98	18	56	22	19
33 Business and administration associate professionals	2388	7.7	44.9	73	21	53	23	13
34 Legal, social, cultural and related associate professionals	1389	4.5	42.5	65	8	51	24	17
41 General and keyboard clerks	796	2.6	46.5	59	8	52	33	12
42 Customer services clerks	842	2.7	43.5	61	13	47	23	13
43 Numerical and material recording clerks	492	1.6	44.0	69	11	50	25	12
44 Other clerical support workers	290	0.9	44.5	58	9	50	23	14
51 Personal service workers	1065	3.4	40.5	23	6	36	18	13
52 Sales workers	2134	6.9	38.8	27	5	41	17	13
53 Personal care workers	3986	12.9	44.5	12	3	51	26	20
81 Stationary plant and machine operators	360	1.2	42.8	18	23	47	20	18
91 Cleaners and helpers	1411	4.6	46.0	10	1	47	25	20
94 Food preparation assistants	374	1.2	46.8	10	2	49	26	20
** All**	30,873	100.0	43.5	63	25	53	22	14

^*^At least one sickness absence day

### Statistical methods

We used StataSE version 14 for analysis [33]. First, we calculated means for all outpatient care visits and the percentage of persons who had used inpatient care services by occupational groups (1-digit level) and by 20 more detailed occupations (2-digit level).

Second, we used multivariate modelling to study whether the differences between occupational groups and occupations could be explained by sociodemographic factors and health status. We used multivariate modelling to estimate the gross (unadjusted) and net (adjusted) associations of occupation with health care services use. For this purpose, several models were run, each including different sets of variables. In model 1, only occupation and age were included. Next, we added sociodemographic factors (education, income and marital status) and measures for health status (special reimbursement entitlements for medicines and sickness absence) separately in models 2 and 3. In model 4 we controlled for all covariates simultaneously.

The models were run for both outcomes and with both the broader and more detailed occupational classifications, separately for men and women. We first included the categorized occupation variables in the regression models and then used the *contrast* command in Stata in order to recalculate the results as comparisons against the grand mean, i.e. all employees in the model [34, 35]. This procedure was chosen as no occupational class would have been a natural choice for a comparison group. Furthermore, we used two overlapping occupational classifications and we wanted to use a common reference point for them. Using the grand mean as a reference serves this purpose well.

The average number of visits to doctors or nurses in outpatient care services was modelled with negative binomial regression models; thus, the results are shown as incidence rate ratios (IRR). The method can be used for over-dispersed count data, when the conditional variance exceeds the conditional mean [36, 37]. The minimum and maximum values of the number of outpatient visits in the occupational classes are shown in Supplementary table 2. The use of inpatient care services was modelled with logistic regression models, and the results are shown as odds ratios (OR). The statistical significance of the estimates was assessed with 95% confidence intervals. As explained above, the estimates and their confidence intervals are presented in comparison to all employed men or women in the data set, respectively.

The analyses were performed separately for men and women, because of the known differences in occupational distributions [38] and in the prevalence of health service use between Finnish men and women [31].

## Results

### Men

Male employees had an average of 3.8 outpatient care visits (during 2018) (Table 3). At the 1-digit level of occupational groups, managers and professionals had the least outpatient care visits (3.3 visits) and clerical and support workers the most visits (4.4 visits). Almost all occupational groups differed from the average in either direction. In general, the number of visits was lower than average in the upper groups and higher in the lower groups. Controlling for socio-demographic factors and health status only had a small effect on the estimates. Adjusting for health status among health care professionals and personal care workers had the greatest impact.

**Table 3:** Outpatient and inpatient care by occupational group and occupation among male employees

	Outpatient health care (IRR)						Inpatient health care (OR)					
**Occupational group (1-digit level)/** **Occupation (2-digit level, 20 most common occupations)**	No of visits	M1	M2	M3	M4	95% CI	%	M1	M2	M3	M4	95% CI
**All**	3.8	1.00	1.00	1.00	1.00		6.8	1.00	1.00	1.00	1.00	
**1 Managers**	3.3	**0.80**	**0.84**	**0.84**	**0.86**	(0.80–0.92)	7.1	0.90	0.93	0.97	0.96	(0.77–1.19)
13 Production and specialised services managers	3.5	**0.85**	**0.89**	**0.89**	**0.91**	(0.83–1.00)	7.2	0.92	0.94	0.99	0.97	(0.72–1.30)
**2 Professionals**	3.3	**0.87**	**0.89**	**0.90**	**0.91**	(0.89–0.93)	6.1	**0.89**	**0.92**	0.94	0.94	(0.87–1.02)
21 Science and engineering professionals	3.0	**0.80**	**0.82**	**0.84**	**0.84**	(0.81–0.88)	6.0	**0.88**	0.91	0.93	0.94	(0.82–1.07)
22 Health professionals	3.5	0.97	1.00	0.97	0.98	(0.89–1.07)	6.4	0.94	0.97	0.97	0.95	(0.69–1.32)
23 Teaching professionals	3.4	**0.85**	**0.86**	**0.88**	**0.88**	(0.83–0.93)	8.0	1.06	1.07	1.11	1.10	(0.92–1.33)
24 Business and administration professionals	3.7	0.95	0.97	0.97	0.97	(0.90–1.05)	5.7	0.80	0.82	0.83	0.82	(0.63–1.07)
25 Information and communications technology professionals	3.5	0.96	0.98	0.98	0.98	(0.94–1.03)	5.2	**0.83**	0.85	0.87	0.88	(0.73–1.05)
**3 Technicians and associate professionals**	3.9	**1.04**	**1.04**	**1.03**	**1.03**	(1.00–1.06)	6.8	1.01	1.02	1.01	1.01	(0.92–1.12)
31 Science and engineering associate professionals	3.7	0.99	0.99	**0.99**	0.97	(0.93–1.02)	6.3	0.94	0.95	0.94	0.95	(0.80–1.12)
32 Health associate professionals	4.6	**1.25**	**1.24**	**1.18**	**1.16**	(1.05–1.29)	9.1	**1.47**	**1.51**	1.34	**1.38**	(1.02–1.86)
33 Business and administration associate professionals	4.1	**1.06**	**1.07**	**1.08**	**1.08**	(1.02–1.14)	6.8	0.96	0.96	0.99	0.98	(0.82–1.18)
34 Legal, social, cultural and related associate professionals	3.8	1.03	1.03	0.98	1.01	(0.91–1.11)	7.7	1.18	1.19	1.12	1.15	(0.84–1.57)
**4 Clerical support workers**	4.4	**1.17**	**1.18**	**1.09**	**1.12**	(1.04–1.20)	7.7	1.16	1.15	1.09	1.10	(0.88–1.39)
**5 Service and sales workers**	3.9	**1.08**	**1.07**	**1.06**	**1.08**	(1.04–1.13)	7.1	1.13	1.10	1.09	1.09	(0.95–1.25)
51 Personal service workers	3.8	0.98	0.98	0.98	1.01	(0.93–1.09)	8.1	1.22	1.19	1.18	1.20	(0.94–1.53)
52 Sales workers	3.5	1.00	1.00	1.00	1.03	(0.96–1.09)	6.3	1.04	1.01	1.03	1.03	(0.83–1.28)
53 Personal care workers	4.6	**1.26**	**1.25**	**1.19**	**1.22**	(1.10–1.35)	7.9	1.24	1.22	1.13	1.15	(0.82–1.62)
54 Protective services workers	4.7	**1.30**	**1.27**	**1.26**	**1.25**	(1.12–1.38)	6.9	1.15	1.12	1.05	1.05	(0.74–1.50)
**6 Skilled agricultural, forestry and fishery workers**	3.9	1.02	1.03	0.95	0.99	(0.78–1.26)	6.3	0.86	0.85	0.79	0.80	(0.35–1.86)
**7 Craft and related trades workers**	4.2	**1.13**	**1.09**	**1.12**	**1.09**	(1.05–1.13)	6.8	1.04	1.01	1.01	1.00	(0.89–1.13)
71 Building and related trades workers, excluding electricians	4.0	**1.12**	**1.07**	**1.11**	**1.09**	(1.03–1.14)	6.2	0.97	0.93	0.95	0.92	(0.77–1.11)
72 Metal, machinery and related trades workers	4.8	**1.27**	**1.23**	**1.25**	**1.23**	(1.15–1.31)	7.9	1.18	1.15	1.14	1.14	(0.93–1.39)
74 Electrical and electronic trades workers	3.8	1.04	1.01	1.02	1.00	(0.93–1.09)	6.1	0.97	0.96	0.93	0.95	(0.72–1.25)
**8 Plant and machine operators, and assemblers**	4.3	**1.11**	**1.08**	**1.07**	**1.05**	(1.01–1.10)	8.2	**1.24**	**1.19**	**1.17**	**1.15**	(1.01–1.31)
81 Stationary plant and machine operators	5.2	**1.36**	**1.34**	**1.30**	**1.28**	(1.20–1.37)	9.2	**1.39**	**1.35**	**1.28**	**1.27**	(1.02–1.56)
83 Drivers and mobile plant operators	3.7	0.98	**0.94**	0.95	**0.93**	(0.88–0.98)	7.8	**1.18**	1.12	1.13	1.11	(0.93–1.32)
**9 Elementary occupations**	4.0	**1.10**	**1.09**	1.05	1.07	(0.99–1.15)	5.3	0.83	0.81	0.78	0.78	(0.60–1.03)
93 Labourers in mining, construction, manufacturing and transport	4.7	**1.30**	**1.27**	**1.23**	**1.23**	(1.12–1.35)	5.2	0.82	0.80	0.74	0.75	(0,53–1.07)

M1: occupational group/occupation + age

M2: M1 + education + income + marital status

M3: M1 + chronic diseases + sickness absence

M4: M1 + education + income + marital status + chronic diseases + sickness absence

Coefficients in bold differ statistically significantly from the reference groups (all male employees); confidence intervals shown only for model 4

Model 4 shows the incidence rate ratios (IRR) compared to all male employees, using occupational groups and occupations separately as independent variables, when all socio-demographic factors and health status were adjusted for. Managers and professionals had a statistically significantly lower number of visits than male employees on average. Among technicians and associate professionals, health associate professionals had a clearly higher IRR (1.16) than other occupations in this group. Clerical support workers had the highest number of visits at the 1-digit level. Among service and sales workers, personal care workers and protective service workers had the highest number of visits. Skilled agricultural, forestry and fishery workers did not differ from all employees. At the 1-digit level, craft and related trades workers and plant and machine operators and assemblers had a higher IRR, but there were some differences at the 2-digit level: the number of visits was higher especially among metal, machinery and related trades workers and stationary plant and machine operators, and lower among drivers and mobile plant operators. Elementary occupations did not differ from all employees, but within this occupational group, labourers in mining, construction, manufacturing and transport had a higher IRR (1.22).

6.8% of male employees had been in inpatient care. Between occupational groups, the proportion varied between 5.3% in elementary occupations and 8.2% among plant and machine operators and assemblers. Controlling for covariates attenuated the differences in some occupations. Controlling for health status had a greater effect, especially among health associate professionals and personal care workers. In the fully adjusted model at the 1-digit level, plant and machine operators and assemblers had higher odds (OR = 1.24) compared to all male employees. At the 2-digit level, the odds were higher than average among health associate professionals and stationary plant and machine operators.

### Women

On average, female employees had 5.7 outpatient health care visits during 2018 (Table 4). In the 1-digit level occupational groups, the number of visits varied between 4.0 (skilled agricultural, forestry and fishery workers) and 6.4 (elementary occupations). The effect of adjustments for sociodemographic factors and health status varied between occupational classes and occupations. In most occupations, sociodemographic factors had a greater effect than health status. The overall biggest effects were found among professional occupations, science and engineering associate professionals, personal care workers, and cleaners and helpers. The results from the fully adjusted model were quite similar to the results found among men. The biggest difference was the less frequent use of health care among craft and related trades workers. Other notable differences compared to men were the more common use among science and engineering associate professionals and sales workers and the less common use among personal service workers. Among clerical support workers, customer service clerks had the highest number of visits.

**Table 4:** Outpatient and inpatient care by occupational group and occupation among female employees

	Outpatient health care (IRR)						Inpatient health care (OR)					
**Occupational group (1-digit level)/Occupation (2-digit level, 20 most common occupations)**	No of visits	M1	M2	M3	M4	95% CI	%	M1	M2	M3	M4	95% CI
**All**	5.7	1.00	1.00	1.00	1.00		8.4	1.00	1.00	1.00	1.00	
**1 Managers**	4.8	**0.81**	**0.89**	**0.87**	**0.90**	(0.82–0.99)	7.3	0.78	0.81	0.84	0.82	(0.56–1.18)
**2 Professionals**	5.0	**0.88**	**0.94**	**0.92**	**0.95**	(0.93–0.97)	7.4	**0.88**	**0.91**	**0.94**	0.93	(0.86–1.01)
21 Science and engineering professionals	4.5	**0.81**	**0.86**	**0.85**	**0.88**	(0.83–0.94)	8.0	1.01	1.05	1.11	1.11	(0.88–1,39)
22 Health professionals	4.7	**0.82**	**0.90**	**0.86**	**0.90**	(0.85–0.96)	7.4	0.88	0.92	0.94	0.92	(0.75–1.14)
23 Teaching professionals	5.1	**0.89**	**0.94**	**0.93**	**0.95**	(0.92–0.98)	7.6	**0.89**	0.92	0.95	0.95	(0.84–1.07)
24 Business and administration professionals	5.0	**0.88**	**0.94**	**0.93**	0.95	(0.90–1.01)	7.3	0.87	0.90	0.93	0.92	(0.74–1.14)
25 Information and communications technology professionals	5.3	0.95	1.02	0.96	1.00	(0.91–1.10)	8.0	1.00	1.03	1.06	1.05	(0.74–1.48)
26 Legal, social and cultural professionals	5.6	1.00	1.05	1.03	1.06	(1.00–1.12)	6.1	**0.72**	**0.75**	**0.74**	**0.74**	(0.58–0.95)
**3 Technicians and associate professionals**	6.0	**1.05**	**1.06**	**1.03**	**1.04**	(1.02–1.06)	8.8	1.06	**1.08**	1.04	1.05	(0.98–1.13)
31 Science and engineering associate professionals	5.9	1.05	1.07	**1.12**	**1.13**	(1.04–1.22)	9.4	1.18	1.19	1.25	1.25	(0,95–1.65)
32 Health associate professionals	6.0	**1.06**	**1.09**	1.02	**1.04**	(1.01–1.08)	9.4	**1.16**	**1.19**	**1.11**	**1.13**	(1.01–1.26)
33 Business and administration associate professionals	6.0	1.04	1.04	**1.04**	**1.04**	(1.00–1.08)	7.5	**0.86**	0.87	0.88	0.89	(0.76–1.03)
34 Legal, social, cultural and related associate professionals	5.8	1.03	1.01	1.00	0.99	(0.94–1.04)	9.6	**1.20**	**1.20**	1.14	1.16	(0.97–1.38)
**4 Clerical support workers**	6.3	**1.09**	**1.06**	**1.09**	**1.07**	(1.03–1.12)	8.8	1.03	1.03	1.02	1.04	(0.90–1.19)
41 General and keyboard clerks	6.2	1.07	1.04	1.04	1.03	(0.96–1.10)	9.8	1.13	1.12	1.10	1.12	(0.88–1.41)
42 Customer services clerks	6.5	**1.15**	**1.11**	**1.15**	**1.14**	(1.06–1.21)	8.6	1.03	1.03	1.03	1.04	(0.82–1.33)
43 Numerical and material recording clerks	6.1	1.06	1.05	1.07	1.07	(0.98–1.17)	8.3	0.99	1.00	1.00	1.02	(0.74–1.40)
44 Other clerical support workers	6.0	1.04	1.00	1.06	1.04	(0.92–1.17)	7.2	0.84	0.85	0.84	0.86	(0.55–1.35)
**5 Service and sales workers**	6.0	**1.07**	1.01	**1.04**	1.01	(0.98–1.03)	8.6	1.06	1.02	1.02	1.01	(0.92–1.10)
51 Personal service workers	5.2	**0.93**	**0.88**	0.95	**0.92**	(0.87–0.98)	8.3	1.05	1.02	1.06	1.06	(0.84–1.32)
52 Sales workers	6.0	1.10	1.04	**1.11**	**1.08**	(1.03–1.13)	6.7	0.88	0.85	0.88	0.88	(0.74–1.05)
53 Personal care workers	6.2	1.09	1.01	1.03	0.99	(0.95–1.02)	9.8	**1.18**	1.11	1.09	1.07	(0.95–1.21)
**6 Skilled agricultural, forestry and fishery workers**	4.0	**0.74**	**0.71**	0.80	0.79	(0.61–1.02)	7.6	0.99	0.98	1.10	1.12	(0.45–2.80)
**7 Craft and related trades workers**	4.1	**0.76**	**0.73**	**0.78**	**0.77**	(0.69–0.86)	6.8	0.85	0.83	0.89	0.88	(0.58–1.34)
**8 Plant and machine operators, and assemblers**	6.1	**1.09**	1.06	1.07	1.05	(0.97–1.14)	11.6	**1.45**	**1.42**	**1.42**	**1.41**	(1.09–1.81)
81 Stationary plant and machine operators	7.0	**1.25**	**1.22**	**1.22**	**1.21**	(1.09–1.34)	12.5	**1.60**	**1.57**	**1.56**	**1.54**	(1.12–2.13)
**9 Elementary occupations**	6.4	**1.10**	1.02	**1.06**	1.03	(0.98–1.08)	9.3	1.06	1.02	0.99	0.99	(0.83–1.18)
91 Cleaners and helpers	6.5	**1.11**	1.03	**1.07**	1.04	(0.98–1.10)	9.3	1.07	1.01	0.99	0.99	(0.81–1.21)
94 Food preparation assistants	5.9	1.00	0.92	0.98	0.94	(0.85–1.05)	9.9	1.14	1.08	1.07	1.07	(0.75–1.53)

M1: occupational group/occupation + age

M2: M1 + education + income + marital status

M3: M1 + chronic diseases + sickness absence

M4: M1 + education + income + marital status + chronic diseases + sickness absence

Coefficients in bold differ statistically significantly from the reference groups (all female employees); confidence intervals shown only for model 4

Among female employees, 8.4% had been in inpatient care. Occupational groups at the 1-digit level ranged between 6.8% (craft and related trades workers) and 11.6% (plant and machine operators and assemblers). Again, the results were very similar to those seen among men. The effects of controlling for sociodemographic factors and health status were for the most part small. After adjustments, the odds for health associate professionals and plant and machine operators and assemblers and for more detailed stationary plant and machine operators remained high.

## Discussion

### Main findings

Occupational groups differed in their use of outpatient and inpatient health care services among working-age employees. However, there were also differences within occupational groups, examined at a more detailed level of occupations. Controlling for covariates explained only part of the differences, more among women than among men. After adjusting for sociodemographic factors and health status, the use of outpatient care services was most frequent among clerical support workers. The biggest difference between men and women was observed among craft and related trades workers, among whom the use of outpatient care services was high among men but low among women. The use of inpatient care services was highest among plant and machine operators and assemblers. At a more detailed level, both adjusted outpatient and inpatient care were more common among health associate professionals and stationary plant and machine operators, among men as well as among women. In addition, outpatient care was common among such groups as business and administration associate professionals, male personal care workers and female sales workers.

### Interpretation of the results

Taking into account both primary and specialized care, the use of outpatient care services seemed to be more common in occupations lower in the socio-economic hierarchy, especially among men. However, there was also variation within the 2-digit level occupations in the same occupational group. Earlier studies on outpatient health care services use by occupational class have mostly studied primary and specialized care separately. These studies have found, on one hand, contradictory results concerning the use of primary care and, on the other hand, more frequent use of specialized care in the higher socio-economic classes. Differences in results concerning primary care may be explained by differences in, for example, study populations, occupational class classifications or health care systems. To our knowledge, there are no prior studies on outpatient health care services use at the level of more detailed occupations.

Concerning inpatient care, our study showed that the use of inpatient care services was most common among stationary plant and machine operators as in a previous study [24]. We also found that health associate professionals had a higher use of inpatient care services, a result that was not obtained in a study by Kaila-Kangas et al. [24] but was instead supported by a study by Varje et al. [25]. However, earlier results [24] indicating a more common use of inpatient care services among male labourers in mining, construction, manufacturing and transport and among female craft and related trades workers and cleaners and helpers, were not supported in our study. In recent years, the number of outpatient visits in specialist care has increased while the number of inpatient care periods has decreased. Inpatient days have also decreased in primary care [39]. This is of significance when comparing the results of this study with previous studies, but does not give rise to concern as to the comparison between the different occupations.

In outpatient care, adjustment for socio-economic factors by and large had a greater effect among women on the estimates than did health status, whereas among men the effects of all adjustments were very small. In inpatient care, adjusting for health status had a greater effect on the estimates concerning the occupational groups. Sociodemographic factors may be assumed to be of lesser importance in terms of inpatient care, since data on hospitalizations include only the most severe cases of illness and exclude less serious cases not requiring hospitalization. Outpatient care visits indicate milder morbidity – accordingly, non-health-related factors can be expected to play a greater role in the use of these services. At the 2-digit level, especially among health associate professionals and male personal care workers, the adjustment of health status had greater effect to estimates than that of sociodemographic factors.

Higher socio-economic groups may be better informed about health and health care and more able to navigate the health care system and communicate with health care professionals [40]. This may increase health care services use in managers and professionals. However, according to our results, their use of health care services was at an average or below average level. A better ability to identify diseases that require care may explain the high use of services among health associate professionals and male personal care workers. Furthermore, high interests in taking care of one’s health and positive attitudes towards health services may explain some differences. Women use health care more frequently than men, and men working in female dominated sectors such as health care may adopt that habit [13]. Respectively, working in a male dominated sector can explain the lower use among female craft and related trades workers and science and engineering professionals.

Differences in outpatient care visits may also be related to sickness certification requirements. In manual occupations, certification is typically required earlier, sometimes even from the first day of illness, than in non-manual occupations [41]. A stricter sickness certificate requirement may increase their number of outpatient visits compared to non-manual workers. In addition, the frequency of mandatory health check-ups may affect differences between occupations. The check-ups are mandatory e.g. for those working in jobs which pose a particular risk of illness or accident [42], and such occupations are mostly manual [10–12].

The Finnish three-sector system is comprised of occupational, private and public health care. First, occupational health care plays a major role in employee health care in Finland [39]. It is the only sector that is free of charge for the patient at the point of delivery and provides easy access to outpatient care. However, the availability and coverage of occupational health care varies across organizations according to the occupational health care contract [14]. There are shortcomings in occupational health care in small enterprises, which are typical in construction, land transport, accommodation and food service activities [15]. This may partly explain the lower use of outpatient care services among female personal service workers and male drivers and mobile plant operators. Second, private sector services are accessible with high co-payments but access to specialist services is easy. Use of private health care services is more common among upper non-manual employees [16] and may thus increase the total number of outpatient care visits in this group. Third, those without access to occupational health care and a low ability to pay turn to the public sector, where patients’ co-payments are low, but access may be difficult and waiting times long [39]. Further studies are needed to better understand the effect of the Finnish three-sector health care system on occupational differences in health care services use.

Both outpatient and inpatient health care was common among male and female health associate professionals and stationary plant and machine operators. For interpretation of the findings, it may be instructive to compare our results to those on mortality [2–4]. The high use of health care services might indicate an overuse of health services; however, it could also indicate genuine demand not fully captured by the health status variables used in our study. Health associate professionals may be better at disease identification and may have a positive attitude towards health care and knowledge of how to use it. Their lower mortality [3] may be another indicator of the same phenomenon. However, they may have some work-related risks such as threat of violence [43], which increases the need of health care services. Stationary plant and machine operators have high risks for chemical and biological hazards [10], occupational diseases [11], work accidents [12] and mortality [2, 4]. They seem to underuse rather than overuse health care services. The severity of symptoms and the influence on working ability can vary by occupation, even if the symptoms are the same. For example, physical disability makes it more difficult to work as a stationary plant operator than as a clerical support worker. The explanations of better ability, attitude and knowledge may also apply to male personal care workers, while the explanations suggesting higher occupational risks may also pertain to male building and related trades workers, to metal, machinery and related trades workers, and to labourers in mining, construction, manufacturing and transport, who have also found to have high mortality [4]. Use of outpatient care services was common also in some other occupations. Female customer service workers have high mortality [4]. Male protective services workers and female sales workers both had high risk of disability retirement but not mortality [3]. The frequent use of outpatient care services among male and female business and administration associate professionals needs further study. The connection between health care use and mortality is thus less clear.

Managers and professionals used health care services less than the average employee. Lower use of health care services might indicate not only better health and a lower need of care but also an underuse of care. However, due to better access to occupational and private health care, better self-rated health [5], a higher probability to work sick and a smaller need of sickness absence certification [41], the underuse does not seem probable. Underuse is more likely among unskilled workers [44]. Among manual workers, male drivers and mobile plant operators, female personal service workers and female craft and related trades workers probably have low access to occupational health care [15] and are thus dependent on the less accessible public health care.

### Strengths and limitations

The key strength of our study was the ability to use individual-level register-based data on all inhabitants in one city. Our study included both outpatient and inpatient care. We were able to cover all three sectors of Finnish outpatient health care: public, private and occupational health care. The last of these is rarely covered in registers [8], which means that previous studies have usually not been able to use register-based data on occupational health care. Because of our access to register sources, we were also able to use more precise information on occupation than many other studies, and adjust for some important correlates of health, which are likely to confound the association between occupation and use of health care services. The register data has practically no missing data and do not suffer from a self-report bias. Thus, they are held to be more reliable than self-reported data on health care services use and socio-economic indicators.

There were also some limitations. The number of health care contacts had to be calculated as separate visits days in outpatient care, because it was not possible to measure each separate visit reliably if there was more than one visit during a single day. Furthermore, as there are few health-related covariates in register data, we were able to utilize somewhat restricted proxy variables for health status. Our data did not include, for example, information on self-reported health or health behaviours, which could affect the use of health services in different occupational groups. The data did not include the causes for visits so we could not estimate how many of the visits were due to a health check-up or common cold and which visits may have been related to a more serious illness or injury. We could not take into account all demand factors such as earlier experience, knowledge, attitudes and preferences, and the supply side, including access to and coverage of occupational health care. Further, we could not take health selection into account. For example, an individual may have previously been in a more hazardous occupation but has switched occupations due to illness. Overall, as our register-based data set included only a restricted set of control variables related to socio-economic status and health, we could not totally explain the occupational-class differences in outpatient and inpatient health care use with our data.

Another limitation is that our data were derived from one city only. The employment rate and the average educational level in Oulu were higher than across all of Finland in year 2018. The share of employees working in industry was somewhat lower and the share of those working in the public sector higher than the country average [27]. The supply of occupational and private sector health care varies by region [39]. Since Oulu is a large city, its population has more opportunities to use occupational health care and private sector health services than inhabitants of more sparsely populated areas. Even though there are some differences in the population composition as compared to the country as a whole, the health care system is fundamentally similar in the whole country. Thus, the results are estimated to be generalizable to the whole of Finland and may provide insights to other countries with roughly similar systems.

## Conclusion

This study brought new information on outpatient and inpatient health care services use by occupation among working-age employees. The use of outpatient and inpatient health care services differs between occupational groups and also within occupational groups by more detailed occupation. Health associate professionals and stationary plant and machine operators frequently used both outpatient and inpatient care services. By and large, the differences cannot be explained by sociodemographic factors and health status.

The explanations for the remaining differences probably vary between occupations. More extensive use of health services may be related to occupational risks that even such extensive use cannot compensate for, or to attitudes and knowledge. Low use of health care services, on the other hand, may be related to, for example, lack of access to occupational health care or overall healthy life habits.

Health-promoting interventions could be targeted at occupations that use health care services more frequently and are known to have high occupational risks. Among high-risk occupations, the health literacy skills could be increased through training, access to health services should be facilitated, and knowledge of services should be increased. Health care services could be made more accessible at the workplace. Health check-ups could be directed at occupations with less frequent use if that is suspected to be due to unmet need. The challenges lie in small enterprises with smaller resources and more limited occupational health care. For managers of companies in high-risk sectors, the usefulness of health services could also be emphasized from the perspective of the employer.

## Supplementary Information

**Additional file 1.** Supplementary Tables 1-2 

## Data Availability

The data sources of this study were the City of Oulu, the Social Insurance Institution of Finland, the National Institute for Health and Welfare, the Finnish Center for Pensions, four large occupational health care providers, Statistics Finland and the Finnish Tax Administration. The data that support the findings of this study are not publicly available because restrictions apply to the availability of these data, which were used under permissions from third-party data holders for the current study. Permissions to obtain register data from these third-party data holders may be applied for scientific research purposes from the Finnish Health and Social Data Permit Authority Findata (https://www.findata.fi/en/). Permission to obtain register data from Statistics Finland may be applied for (https://www.tilastokeskus.fi/meta/tietosuoja/kayttolupa_en.html).

## Abbreviations

IRR: Incidence rate ratios
OR: Odds ratio
