# Supplementary material for: Use of outpatient and inpatient health care services by occupation—a register study of employees in Oulu, Finland
Source: BMC Health Serv Res. 2022 May 3;22:597. doi: 10.1186/s12913-022-07970-y (PMC9066753; doi:10.1186/s12913-022-07970-y)
Supplement: Supplementary file 1 — Additional file 1. Supplementary Tables 1-2 [file 12913_2022_7970_MOESM1_ESM.pdf]

Supplementary Table 1. Use of outpatient health care and inpatient care by sociodemographic factors and health status among employees.

	Men		Women	
	Outpatient**	Inpatient***	Outpatient**	Inpatient***
**Age**				
25-29	3.0	4.5	4.9	6.2
30-34	3.3	4.4	5.3	6.2
35-39	3.4	6.0	5.2	7.6
40-44	3.5	6.3	5.5	7.9
45-49	4.0	6.6	5.7	8.4
50-54	4.4	9.3	6.2	10.0
55-59	4.9	9.9	6.6	9.9
60-64	5.3	12.1	6.6	12.6
**Education***				
Tertiary	3.5	6.3	5.5	8.0
Secondary	4.1	7.2	6.2	9.2
Primary	4.1	8.4	5.8	8.1
**Income***				
Highest quartile	3.6	6.4	5.1	7.6
2nd highest quartile	3.9	6.9	5.6	8.6
2nd lowest quartile	4.2	7.0	6.2	8.6
Lowest quartile	3.9	7.3	6.3	9.1
**Marital status***				
Married	3.7	7.1	5.5	8.4
Unmarried	3.7	5.9	5.7	7.5
Separated/widowed	4.3	7.7	6.8	10.9
**Chronic diseases***				
No	3.4	6.1	5.0	7.1
Yes	5.3	9.6	8.0	12.7
**Sickness absence days***				
0	3.5	6.0	5.0	7.1
1-60	6.7	13.4	8.7	13.4
61-365	9.4	19.9	12.6	22.8
**All employees**	3.8	6.8	5.7	8.4

*Adjusted for age
**Number of visits
***Percentage of persons

Supplementary Table 2. Minimum and maximum values of the number of outpatient visits by occupational class among male and female employees.

	Men	Women
1 Managers	0-41	0-36
2 Professionals	0-145	0-149
3 Technicians and assoaciate professionals	0-147	0-160
4 Clerical support workers	0-46	0-91
5 Service and sales workers	0-118	0-83
6 Skilled agricultural, forestry and fishery workers	0-28	0-34
7 Craft and related trades workers	0-62	0-43
8 Plant and machine operators, and assemblers	0-70	0-51
9 Elementary occupations	0-45	0-85
All employees	0-147	0-160
